# Cartooning in Biological Learning

**DOI:** 10.1002/bmb.70040

**Published:** 2026-02-25

**Authors:** Soukaina Bahsoun, Mhairi Morris, Elizabeth Akam

**Affiliations:** ^1^ School of Sport Exercise and Health Sciences Loughborough University Loughborough UK

## Abstract

Teaching bioscience subjects requires helping students understand complicated and abstract concepts. An effective method of helping students understand these abstract topics is the use of concept cartoons. Concept cartoons are cartoon‐style drawings that illustrate everyday situations. In constructivism, learners create their own knowledge and understanding through exploring and interacting with the external world, reflecting on their experiences and building new knowledge on top of pre‐existing knowledge. Here we demonstrate the effectiveness of using cartooning across three undergraduate biology modules at each level four to six to facilitate active learning. Students constructed and reconstructed their knowledge of genetics, cell signaling and cancer through self‐generated cartoons. Seventy‐nine students took part in the activity, working in teams of 2–6 members and turned a biological process into a cartoon. The evaluation of the activity involved a 10‐question multiple‐choice quiz and an 18‐question survey using a 5‐point scale and free‐form comments. There was a significant statistical difference in performance when comparing cartooned and not cartooned subtopics. Student engagement scored an average of 4.8, learning scored 4.5, and understanding scored 4.2. Sixty eight percent of students stated that they preferred the cartooning activity over learning in a traditional question and answer format. In the free‐form comments, students commonly cited improved understanding as a benefit to this activity. Beyond being a tool to learn the material, the cartooning activity proved beneficial in fostering teamwork among the student cohorts. Overall, cartooning proved to be an effective method in making abstract and complicated topics more approachable to students.

## 1 Introduction

The study of cells covers cell structure and function, molecular components, subcellular structures and function, metabolic processes, genetics and genomics, cell signaling and communication and cell cycle and division [1, 2]. Learning cell biology presents students with several challenges such as complex, acronymized and often historical terminology, abstract concepts, multi‐step processes, integration of knowledge, and volume of information. Memorizing facts and figures is not the most efficient approach to learning and understanding biology; grasping the “interconnectedness and dynamic nature of living systems” is essential [3]. “In fact, memorizing anything discourages deep thinking. Deep thinking is essential because understanding is the residue of thinking” [4]. It has long been acknowledged by biology teachers that there is a need to move away from traditional passive lecturing and instead use innovative, new and multi‐modal teaching approaches to facilitate active, permanent, deep, self‐directed, and student‐centered learning [3, 5, 6, 7, 8, 9]. Interactive teaching and active learning promote engagement, motivation, deep understanding, creative and critical thinking, curiosity, and collaborative skills [3, 9, 10]. The literature is rich in examples of active and interactive approaches that have been used to engage biology students. Some examples include pre‐ and post‐lecture puzzles [7], concept mapping [11], interactive slide presentations [12], peer‐led groups [13], molecular storytelling [14], interactive Metabolism (iM‐tool) [10], escape rooms [15], virtual reality [16, 17], and learning by teaching and peer review [9]. Other strategies suggested by Akhmadkulovna [3] include hands‐on experiments, group discussions, technology‐enhanced learning, problem‐based learning, and case studies. Moreover, flipped teaching (FT) combined with dramatization has been shown to improve student knowledge when used to teach steroid hormone and protein hormone cell signaling in an Animal Physiology course [18].

Concept cartoons, as a learning and teaching strategy, were first introduced by Keogh and Naylor in the late 1990s [19]. Since then, this strategy has been adopted by many researchers and teachers and has proven to be effective in science and biology teaching. Concept cartoons have been generally used to engage learners, generate discussions, illustrate science enquiries, and to create opportunities for creative thinking and applying ideas [20, 21]. Moreover, concept cartoons have been shown to remedy students' misconceptions and improve understanding [22, 23, 24, 25, 26]. Atchia and Gunowa demonstrated that the incorporation of concept cartoons within the conceptual change model proved effective in addressing students' misconceptions; particularly in the topic “plant nutrition” [22]. Izgi and Basar conducted a study to assess the views of 53 senior pre‐service science teachers about the use of concept cartoons in science teaching. The study concluded that the use of concept cartoons positively affects the learning process because they have the potential to “provide long‐lasting learning, avoid misconceptions and improve higher thinking skills” [23]. Moreover, concept cartoons were used by Kusumaningrum and Indriyanti to address students' misconceptions in the topic of buffers. In addition to being remedial, concept cartoons were found to promote active learning, students' motivation, and enquiry skills [24]. Concept cartoons have also been trialed in online teaching and learning, and it was shown that they can be considered by learners an enjoyable and useful learning activity [27].

Teaching and learning materials, including concept cartoons, are commonly provided to the students. However, the value of teaching materials can be significantly enhanced if they are created by the students, tailored to their needs and benefits, and centered around the students' perspectives [28]. This framework aligns with a learner‐centered pedagogical approach where students actively participate in the creation and use of educational resources, hence fostering a sense of ownership and improving understanding [29]. Without doubt, when using this constructivist approach, students and teachers must work in partnership. “Students need to put forth the effort necessary to develop their knowledge and skills and institutions need to provide the appropriate environment to facilitate student learning” [30]. In this study, we designed seminars to provide students with an opportunity to construct and reconstruct their knowledge of fundamental cellular biological concepts through self‐generated cartoons. By creating their own cartoons, students across three undergraduate biology modules at each level four to six were invited to critically analyze the subject matter, identify key misconceptions, and translate complex ideas into accessible visual representations. The study had three main aims:

1. To engage every student in the content beyond the first level of learning in Bloom's taxonomy.
2. To give every student the chance to engage with the content, with an emphasis on building knowledge and skills.
3. To create a memorable learning experience with long‐lasting impact using a student‐centered approach.

## 2 Methods

This study was conducted in partnership with students enrolled in three undergraduate modules (courses) at different stages of their degree—equivalent to first, second and third year (UK levels 4–6) to facilitate active learning and knowledge building of a complicated biology topic (Table 1). The students were or are still enrolled in one of the following programs (degrees) at Loughborough University: Biological Sciences, Human Biology and Natural Sciences. Seventy‐nine students in total took part in the study.

**TABLE 1:** List of modules, levels and titles and topics chosen for the cartooning activity.

Level	Module title	Topic chosen
Part A (first year—level 4)	Genetics and molecular biology	DNA repair mechanisms
Part B (second year—Level 5)	Cellular signaling and transport	Signaling life and death
Part C (third year—level 6)	Biology and oncology	Molecular hallmarks of cancer

Table 1 lists the modules' levels, titles, and the topic chosen for cartooning in each module. The choice of topics was based on the following criteria: complicated, multistep processes and where a topic can be divided into subtopics.

Students were introduced to the topics and the subtopics of their module in a typical lecture. A few days after the lecture, students attended a seminar where the cartooning activity was conducted. To initiate the session, students took a brief multiple‐choice quiz (mainly recall questions) to refresh their memory and create an engaging learning atmosphere. The quiz was conducted using Vevox, an online interactive polling platform.

**FIGURE 1:**
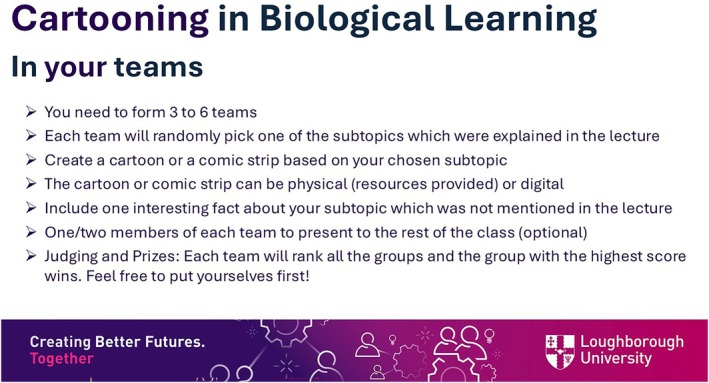
Instructions of the cartooning activity shared with the students.

**FIGURE 2:**
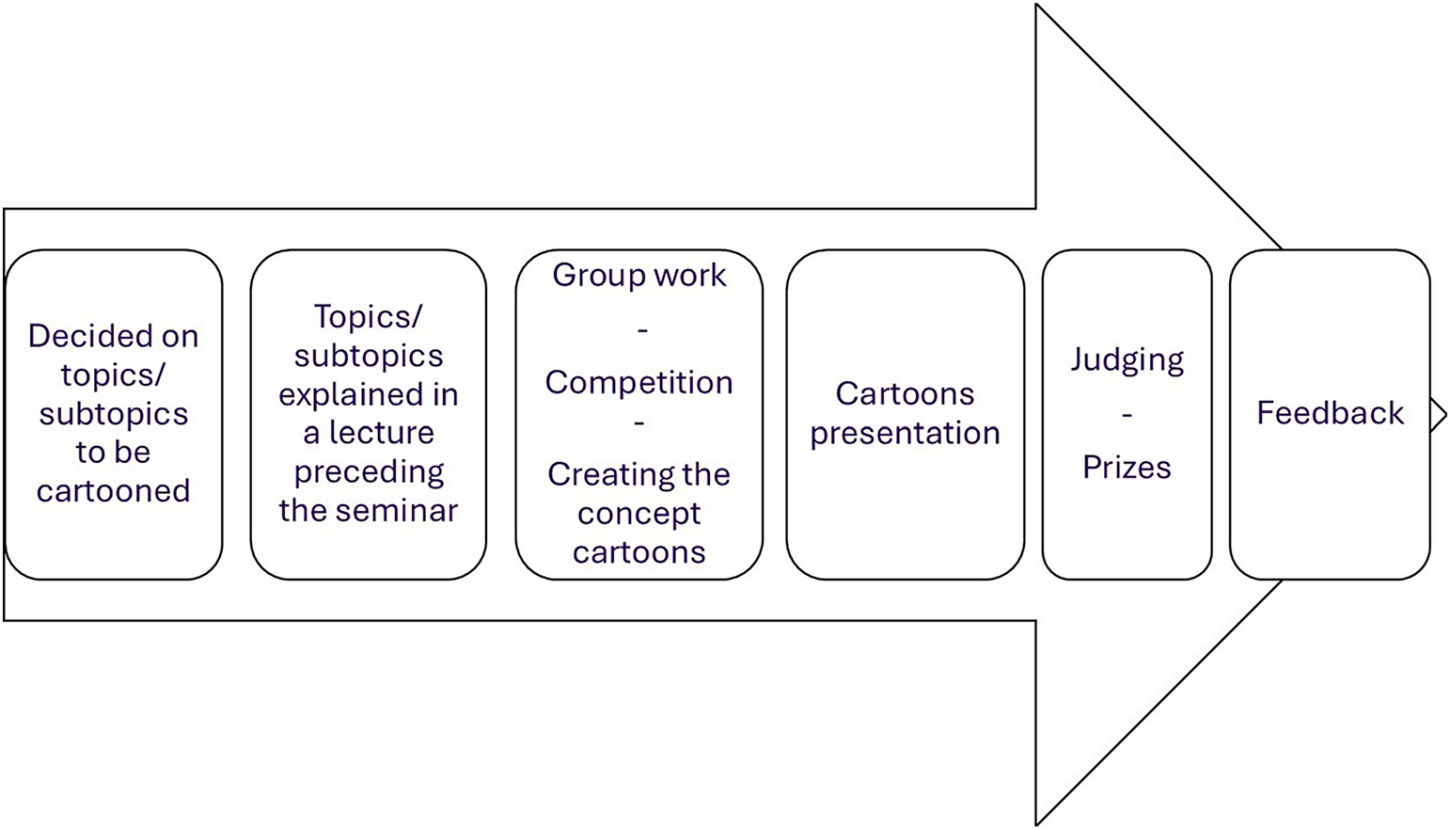
Summary of the seminar design.

After the quiz, students were introduced to the cartooning activity. First, students had to form teams of two to six members. Then, the instructions of the activity were shared (Figure 1). In brief, students had to randomly choose one of the subtopics and create a cartoon based on it. Of note, only three out of the five subtopics were on offer to allow a comparative learning evaluation among cartooned and non‐cartooned subtopics. Students were given the option to present or just share their final visual representation. The activity was run as a friendly competition with chocolate prizes to introduce an element of fun and reward without creating any pressure. In addition, the prospect of winning a small prize and relying on the students to judge and rank the final cartoons was intended to generate a more dynamic and interactive classroom experience. Figure 2 summarizes the seminar design.

Following the presentations, all students reviewed the cartoons produced by each group. A peer‐assessment process was implemented whereby each group ranked the cartoons created by the other groups; self‐ranking in the top position was permitted. Rankings were converted into points, with the higher‐ranked groups receiving more points and lower‐ranked groups receiving fewer points. The points awarded by all the groups were then aggregated to produce a final score for each group. The group with the highest cumulative score was designated as the winner.

To collect evaluation data and feedback on the activity, a survey was created based on published sources [31, 32, 33, 34]. The survey included 18 questions (a mix of questions using a 5‐point scale and free‐form comments). Students filled in the survey anonymously via Microsoft Forms. Collected quantitative data was analyzed using Excel [35] and collected qualitative data was analyzed across questions and modules via inductive thematic analysis following the steps outlined in Maguire & Delahunt [36] and Braun and Clarke [37]. Ethical approval to collect data and to publish examples of students' cartoons was obtained from Loughborough University (2023‐16,192–16,306). In addition, students who participated in the activity gave informed consent to publish their work.

To assess whether students performed differently on subtopics that had been cartooned compared to subtopics that had not been cartooned, we designed a quiz consisting of 10 multiple choice questions. Six questions covered cartooned subtopics, and four questions covered subtopics that were not cartooned. Questions were designed to be comparable in style and level of difficulty. Each of the part A and part B modules administered a distinct quiz relevant to the topic chosen for this activity (Table 1). For each question the average of correct answers was calculated. These question‐level averages across modules were then combined as a single dataset of 20 observations (2 quizzes × 10 questions). In SPSS, each question was categorized as “cartooned” or “not cartooned.” The data was checked for normality and homogeneity of variance using Shapiro–Wilk test and Levene's test respectively. Mann–Whitney U was used to determine the significant difference between cartooned and non‐cartooned subtopics. The difference was deemed significant if *p* ≤ 0.05.

## 3 Results

The evaluation of the cartooning activity involved an 18‐question survey using a 5‐point scale and free‐form comments.

### 3.1 Activity Design and Structure

**FIGURE 3:**
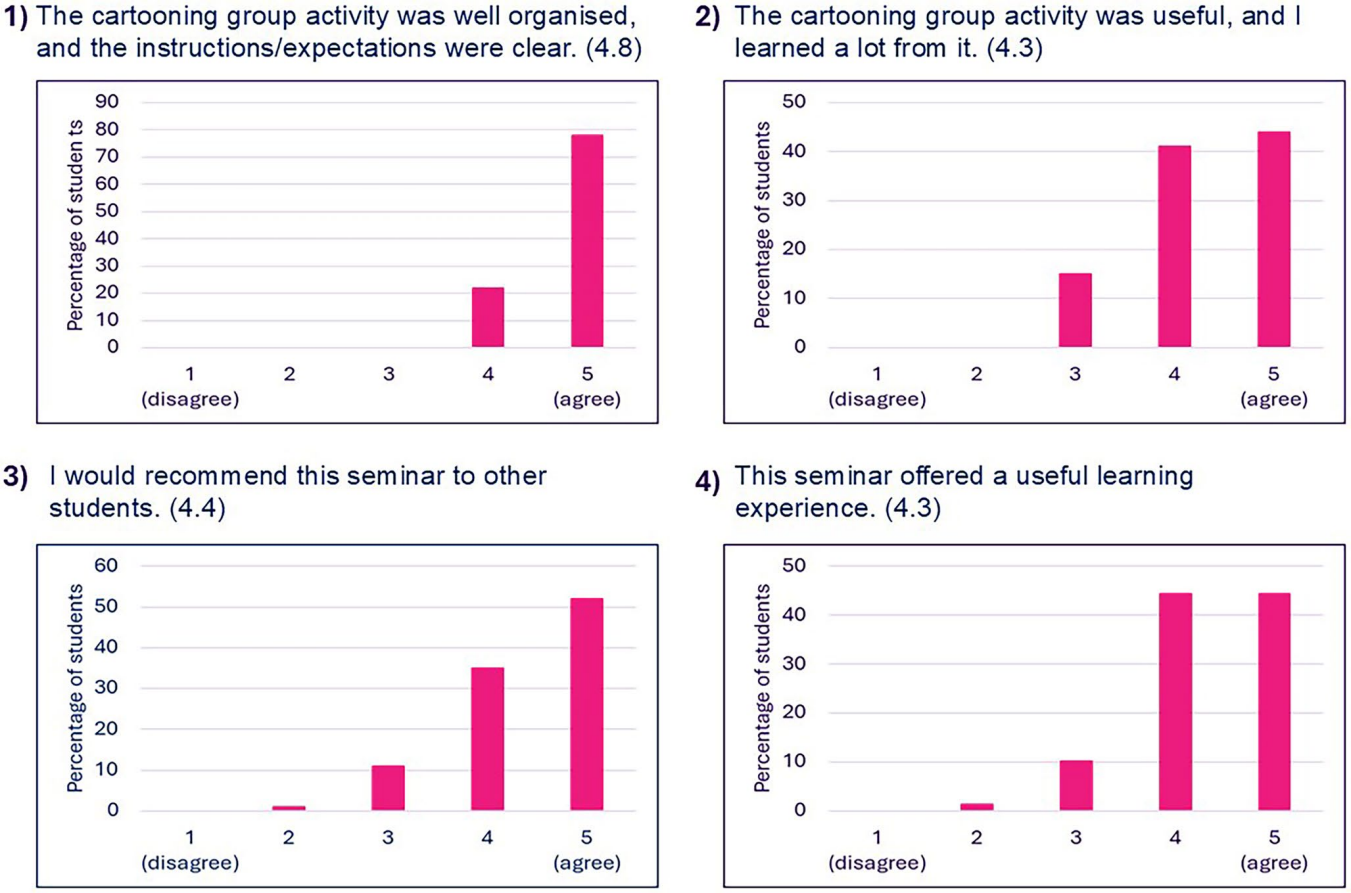
Students' rating of the cartooning activity design and structure. The four questions which were asked are presented above the graphs with numbers in brackets referring to the average score for each question. Data is presented as percentage of students who took part in the activity (total number of students who took part in the activity was 79).

Four questions addressed the cartooning activity design and structure (Figure 3). The average scores for all of these questions were above four, reflecting a predominant trend of agreement in the response set. As shown in Figure 3, 78% of students gave a score of five when asked if the cartooning activity was organized and its instructions were clear; the rest selected four (Question 1—Figure 3). In addition, more than 80% of students gave a score of four or five when asked if the cartooning activity was useful and if they would recommend it to other students (Questions 2, 3 & 4—Figure 3).

### 3.2 Engagement and Participation

**FIGURE 4:**
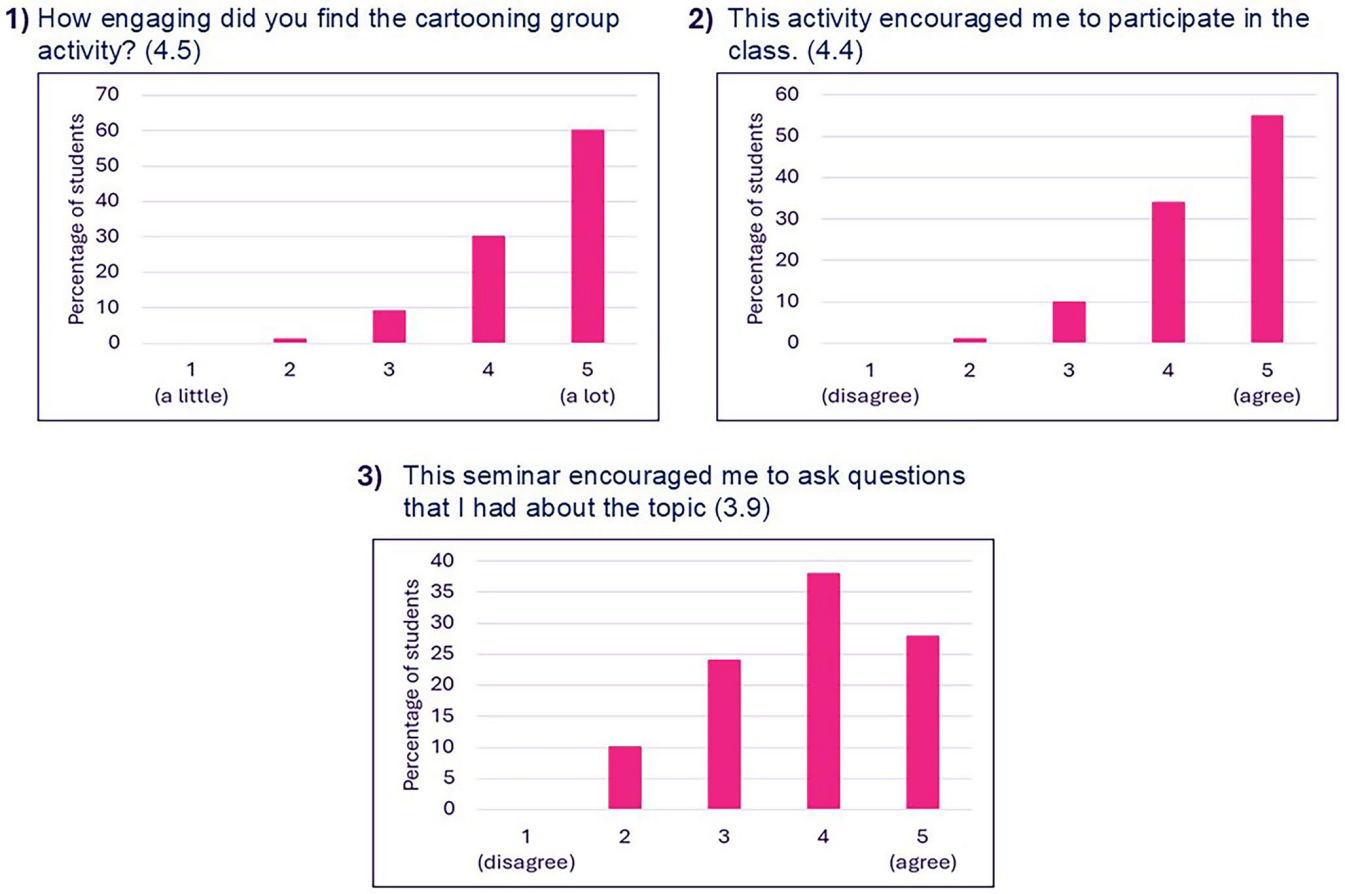
Students' rating of their engagement and participation in the cartooning activity. The three questions which were asked are presented above the graphs with numbers in brackets referring to the average score for each question. Data is presented as a percentage of students who took part in the activity (total number of students who took part in the activity was 79).

Three questions addressed students' engagement and participation in the cartooning activity (Figure 4). Sixty percent and 30% of students gave a score of four and five respectively when asked about how engaging the cartooning activity was, evidencing a strong skew toward agreement (Question 1—Figure 4). Furthermore, 55% and 34% of students gave a score of four and five respectively regarding the cartooning activity being an encouragement to participate in class, reflecting a positive perception (Question 2—Figure 4). The scores to the question “this seminar encouraged me to ask questions that I had about the topic” were more distributed but still skewed toward the higher end of the scale, indicating a predominantly positive response 66% of participants selected either four or five, 24% were neutral (scored three), about 10% selected two indicating a low level of disagreement and no students selected the lowest score of one (Question 3—Figure 4). This highlights the need to provide alternative ways for student to ask questions. For example, allowing students to write down their questions and submit them to the moderator could help include introverted students who may feel reluctant to speak up.

### 3.3 Learning Outcomes

**FIGURE 5:**
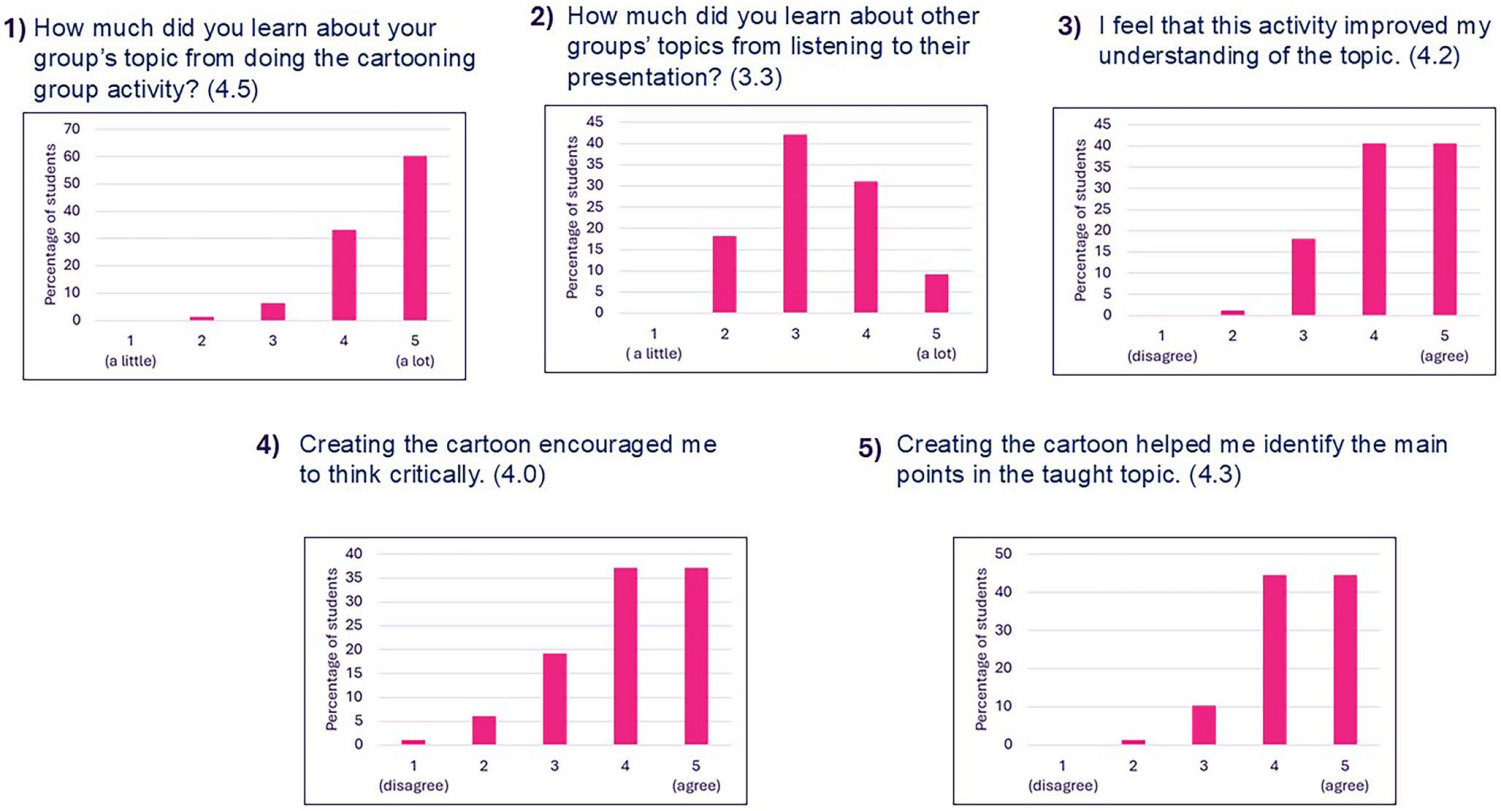
Students' rating of the learning and understanding gained through the cartooning activity. The five questions which were asked are presented above the graphs with numbers in brackets referring to the average score for each question. Data is presented as percentage of students who took part in the activity (total number of students who took part in the activity was 79).

Five questions addressed students' learning and understanding of the topics and subtopics taught via the cartooning activity (Figure 5). Students were asked about how much they learned about their group's topic and other groups' topics (Questions 1 & 2—Figure 5). The vast majority of respondents indicated agreement that they learned a lot about their group's topic, with 60% selecting a score of five and 33% selecting a score of four. The pattern of scores related to learning about other groups' topics was different, with 42% selecting a score of three indicating neutrality and 40% selecting a score of four or five. The other three questions relating to improved understanding, critical thinking, and identifying the main points in the taught topic scored on average 4.2, 4, and 4.3 respectively (Questions 3, 4 & 5—Figure 5). These scores show that there is a clear concentration of responses at the upper end of the scale.

### 3.4 Reflection and Future Use

**FIGURE 6:**
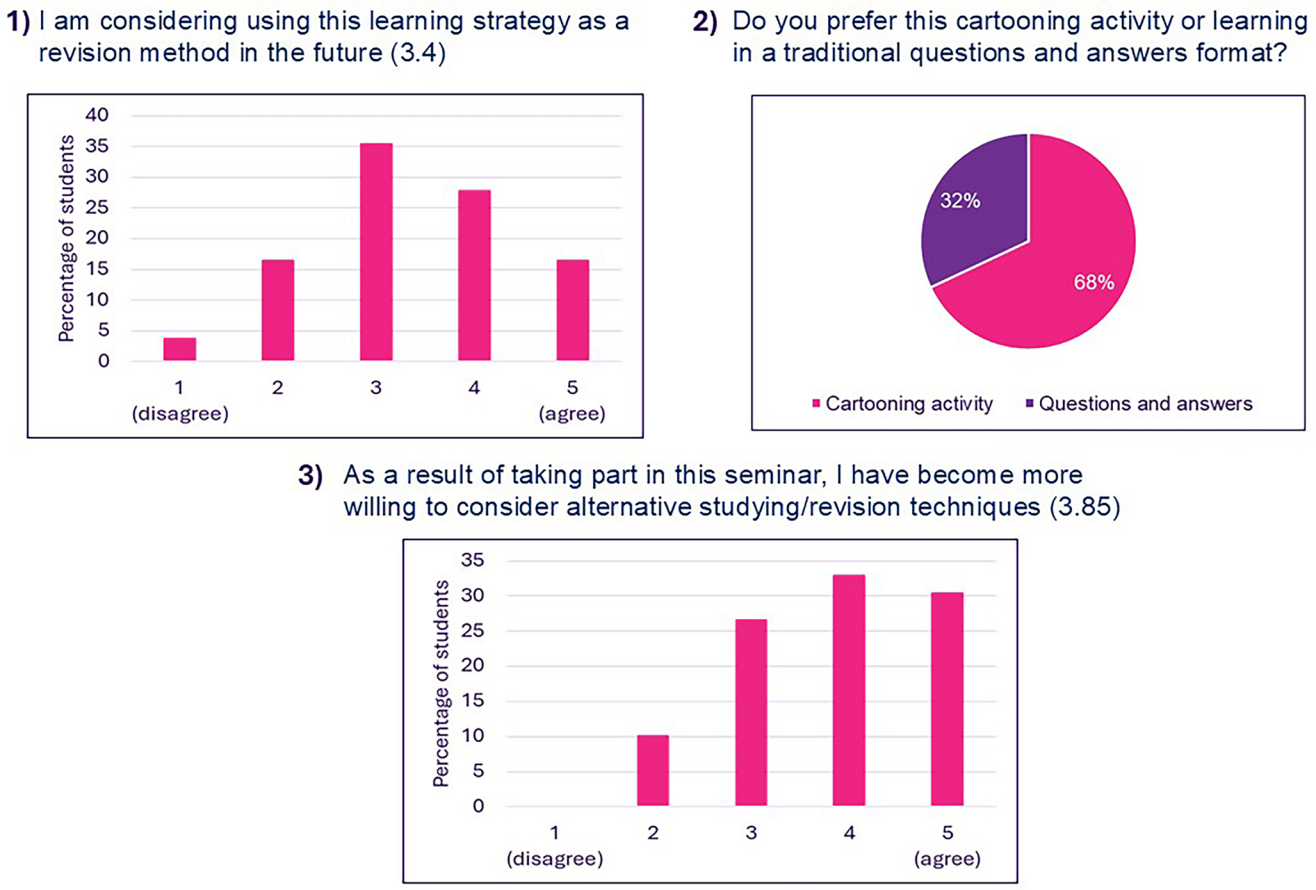
Students' rating of the potential future use of the cartooning activity. The three questions which were asked are presented above the graphs with numbers in brackets referring to the average score for each question. Data is presented as a percentage of students who took part in the activity (total number of students who took part in the activity was 79).

Three questions assessed students' reflections on the cartooning activity and its potential future application (Figure 6). Most students (68%) preferred the cartooning activity over learning in a traditional questions and answers format (Question 2—Figure 6). 44% of students selected a score of four or five when asked whether they will consider using cartooning as a revision strategy in the future compared to 20% who selected a score of one or two. This data shows a mixed response to this question (Question 1—Figure 6). The scores to the question “as a result of taking part in this seminar, I have become more willing to consider alternative studying/revision techniques” show a clear positive trend with a combined 63% of students selecting a score of four or five, 27% selected the neutral midpoint (three) and 10% selected disagreement (two) while no lowest score (one) selected (Question 3—Figure 6).

### 3.5 Assessment of Learning

**FIGURE 7:**
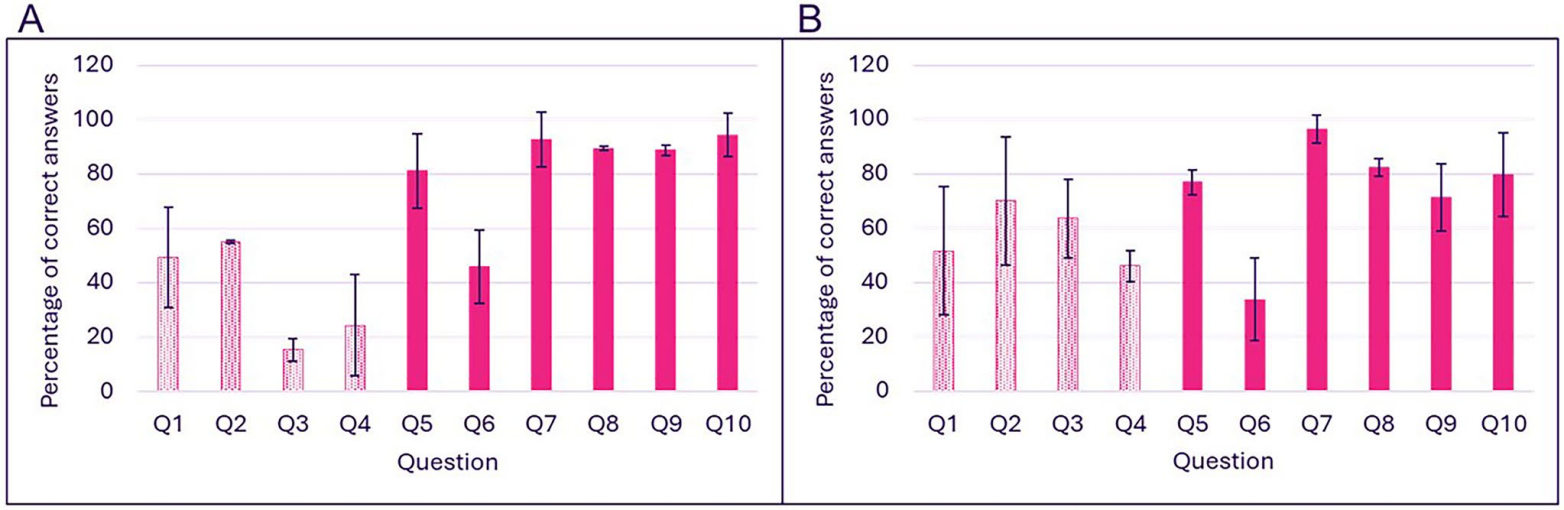
Performance of students on the multiple‐choice quiz which included questions on non‐cartooned subtopics (patterned histograms) and on cartooned subtopics (solid histograms). (A) Graph showing percentage of correct answers in part A module and (B) showing percentage of correct answers in part B module. Data are presented as the average of correct answers across two seminars in each module. Bars represent ±SD.

Students' learning across part A and part B modules was assessed via a quiz composed of 10 multiple choice questions. The percentages of correct answers on subtopics that were cartooned and not cartooned are presented in Figure 7. The question‐level averages across modules were combined as a single dataset of 20 observations. Separate Shapiro–Wilk tests indicated that the data were not normally distributed. Based on the median, Levene's test indicated that the variances were homogeneous. Mann–Whitney U test showed a significant statistical difference in performance when comparing cartooned and non‐cartooned subtopics (U = 12.0, *p* = 0.004, median (IQR) 50.55 (31.69) vs. 81.74 (19.32), respectively).

### 3.6 Open‐Ended Feedback

Three qualitative questions allowed students to include detailed feedback and personal reflections. These questions were:

1. Please comment on how this seminar has changed your attitude toward this subject.
2. Beyond being a tool to learn the module material, were there any other benefits to doing this group activity?
3. Do you have any further comments, feedback, or suggestions about this group activity?

**FIGURE 8:**
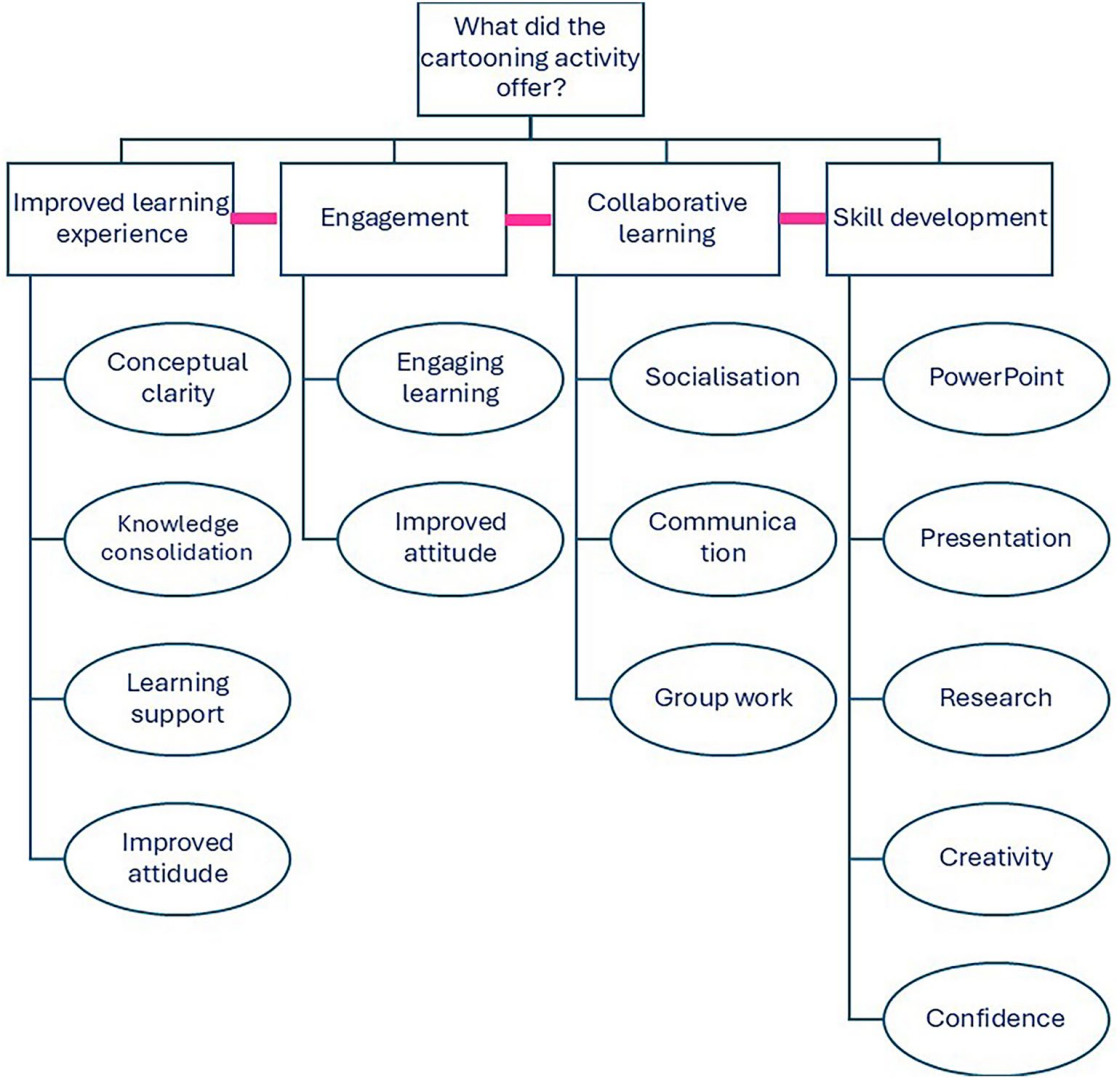
Thematic map: Four themes were identified: Improved learning experience, engagement, collaborative learning and skill development. The subthemes are presented in ovals. The pink lines signify the links among the themes.

**FIGURE 9:**
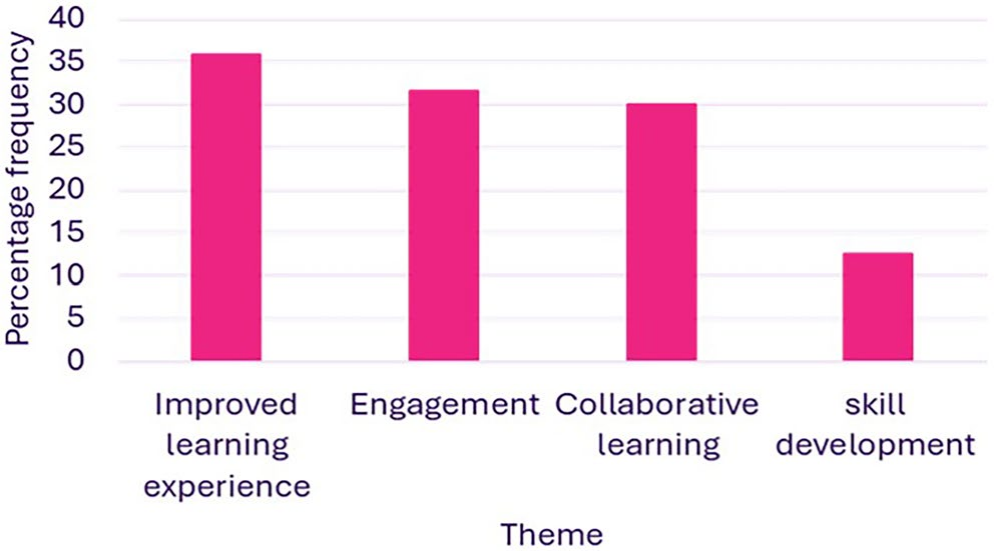
graph showing percentage frequencies of responses associated with each of the four themes identified: Improved learning experience, engagement, collaborative learning and skill development. Percentage frequencies were calculated using the following formula: (number of comments/total number of comments) × 100.

The free‐form answers to these questions were analyzed and the themes and subthemes identified are presented in Figure 8. A total of 120 comments were analyzed, with Figure 9 illustrating the frequency of responses associated with each of the four themes that were identified: Improved learning experience, engagement, collaborative learning, and skill development.

#### 3.6.1 Improved Learning Experience

Overall, 36% of students' responses described aspects related to an improved learning experience achieved via the cartooning activity. In particular, students described conceptual clarity, knowledge consolidation, learning support, and improved attitude. These aspects of improved learning emerged consistently across multiple student responses.

It has simplified complicated processes so it's easier to learn and consolidate.

It has widened my understanding of the signaling pathways and has helped them seem easier to understand in bite sized chunks.

It helped visual learning and broke down a very complicated topic into easier steps.

It has changed my attitude toward the subject, but that is not a bad thing its actually improved my attitude toward the subject area.

#### 3.6.2 Engagement

The free‐form comments revealed a recurring emphasis on engagement. 32% of responses highlighted engaging learning and improved attitude toward the subjects taught. The prevalence of this theme suggests that it was a significant aspect of the student experience.

It makes learning a lot more engaging and breaking down steps makes learning easier.

It made it feel more interesting and engaging whilst also adding an element of fun to it.

More interested and engaged instead of passively listening to the content.

Positively changed my attitude, making me more engaged in the subject.

#### 3.6.3 Collaborative Learning

The theme of collaborative learning appeared mostly in response to the question “beyond being a tool to learn the module material, were there any other benefits to doing this group activity?” 67% of responses to this question (equivalent to 30% of the overall free‐form responses) referred to socialization, communication and group work as benefits to the cartooning activity. Students commented on the importance of getting to know people in the same class, learning from and teaching classmates and collaborating on ideas.

Good to socialize and help to explain misunderstood points to other group members and solidify knowledge.

Learning from others and teaching your peers is an effective and fun revision technique.

Working together and explaining challenging steps to each other if one person doesn't understand.

Socializing and meeting different people on the course.

#### 3.6.4 Skill Development

Skill development was another theme which appeared mostly in response to the question “beyond being a tool to learn the module material, were there any other benefits to doing this group activity?” 24% of responses to this question (equivalent to 12% of overall free‐form responses) described a specific skill and/or confidence and creativity being improved as a benefit of the cartooning activity. Numerous students articulated this theme in their feedback.

Social aspect to learning working as a group creatively.

Got me to research the topic.

Working on public speaking skills and learning scientific communication.

Good fun and developed other creative and presentation skills.

I also learned more about PowerPoint.

### 3.7 Examples of Student‐Created Cartoons

**FIGURE 10:**
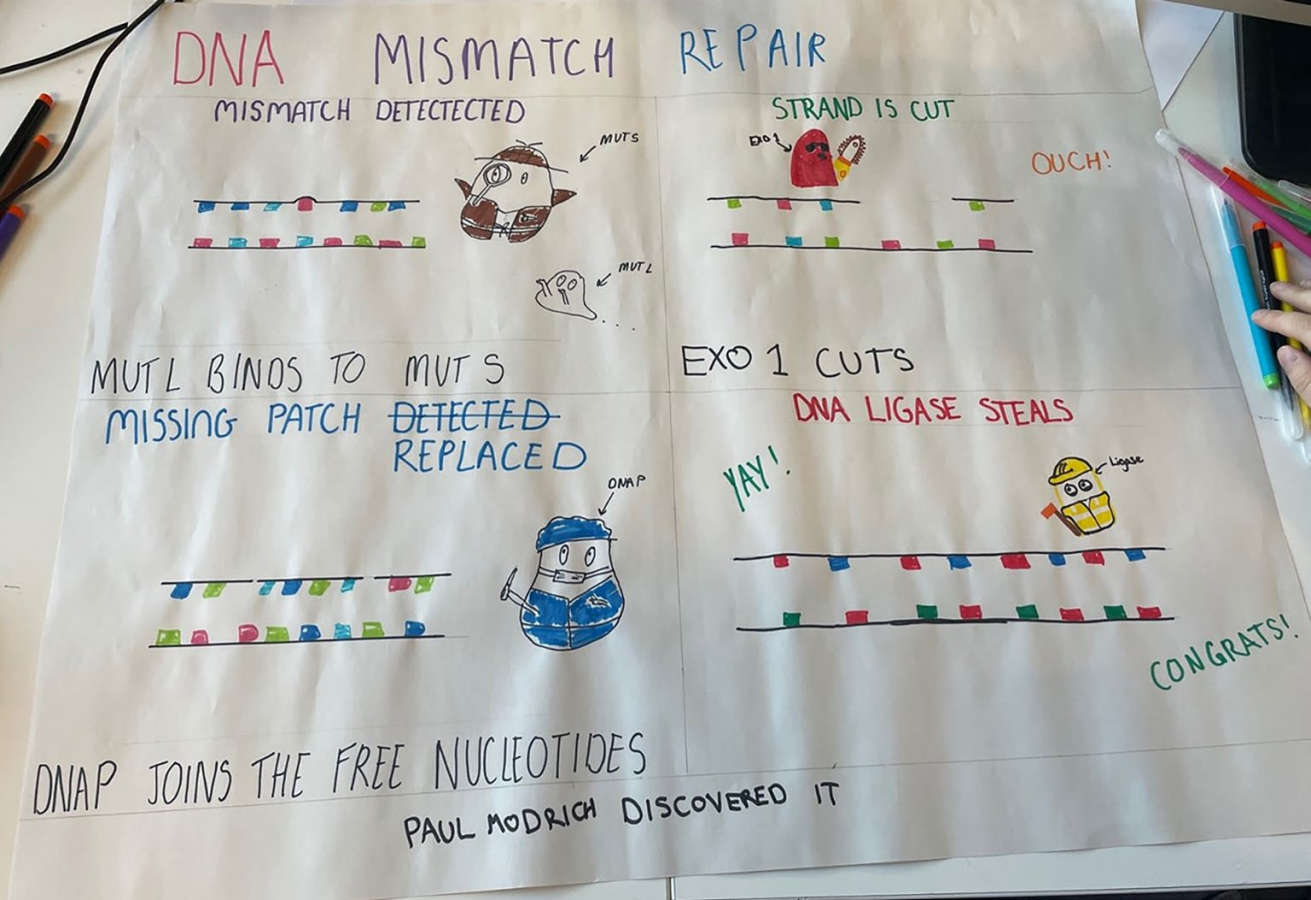
Example of student work producing a concept cartoon about DNA Mismatch Repair (MMR). It shows proteins, like MUTS2 (illustrated as a detective) and MUTL, recognizing mismatched base pairs and initiating the repair process by causing a loop structure which is subsequently cut; “ouch” (excised by exonuclease (EXO‐1)). It then details the role of other key proteins such as DNA polymerase re‐filling the nucleotides and DNA ligase sealing any gaps in the sugar‐phosphate backbone of the molecule.

**FIGURE 11:**
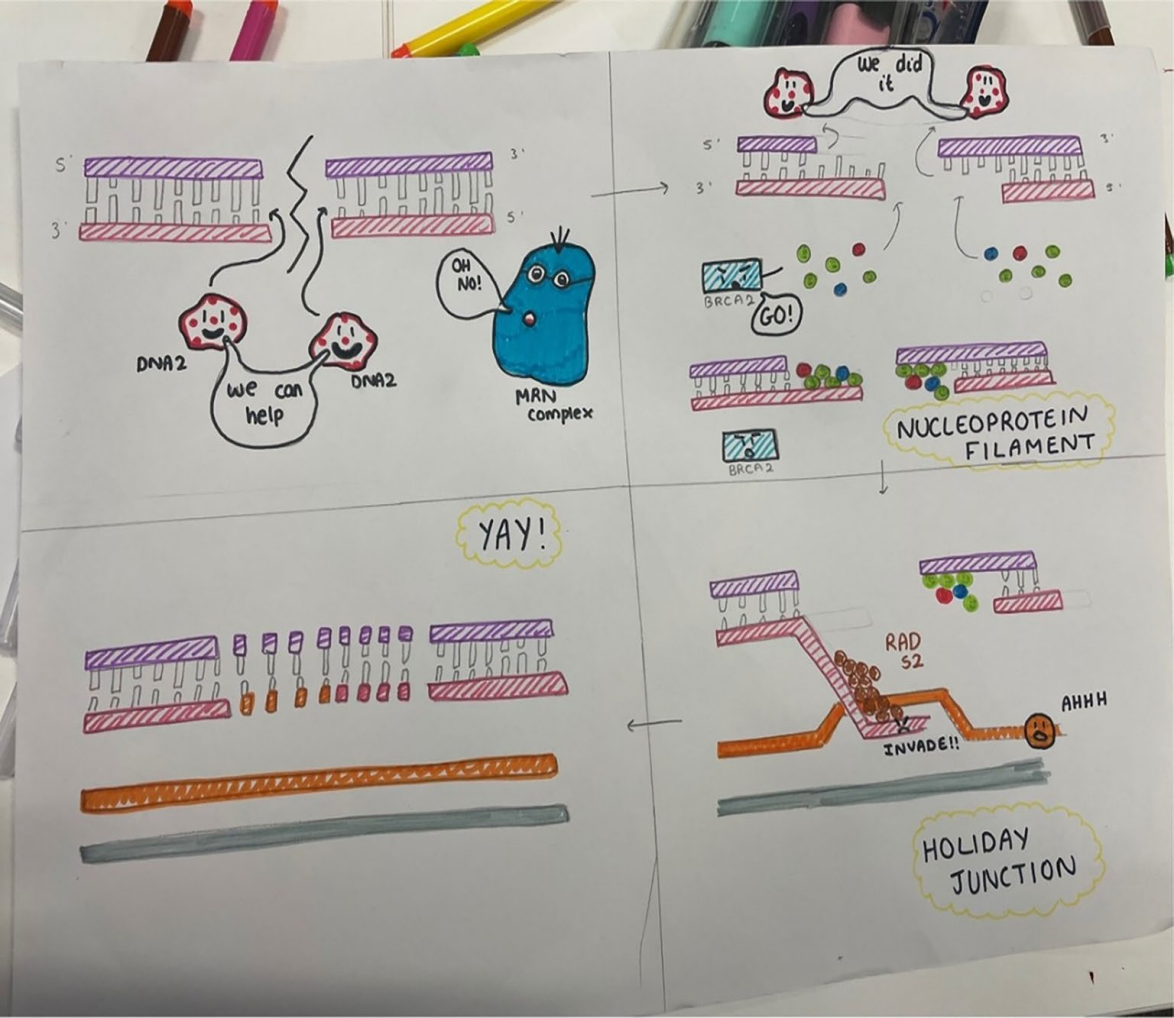
Exemplar of student‐generated concept cartoon illustrating the DNA repair mechanism of Homologous Recombination (HR). After the introduction of a double stranded break (DSB), the ends are recognized by the MRN protein complex (Mre11‐Rad50‐NBS1). This initial step is followed by further protein recruitment (DNA2, BRAC2; cooperativity of system illustrated by “we can help”) to repair the DNA molecule via nucleoprotein filament formation and strand invasion.

**FIGURE 12:**
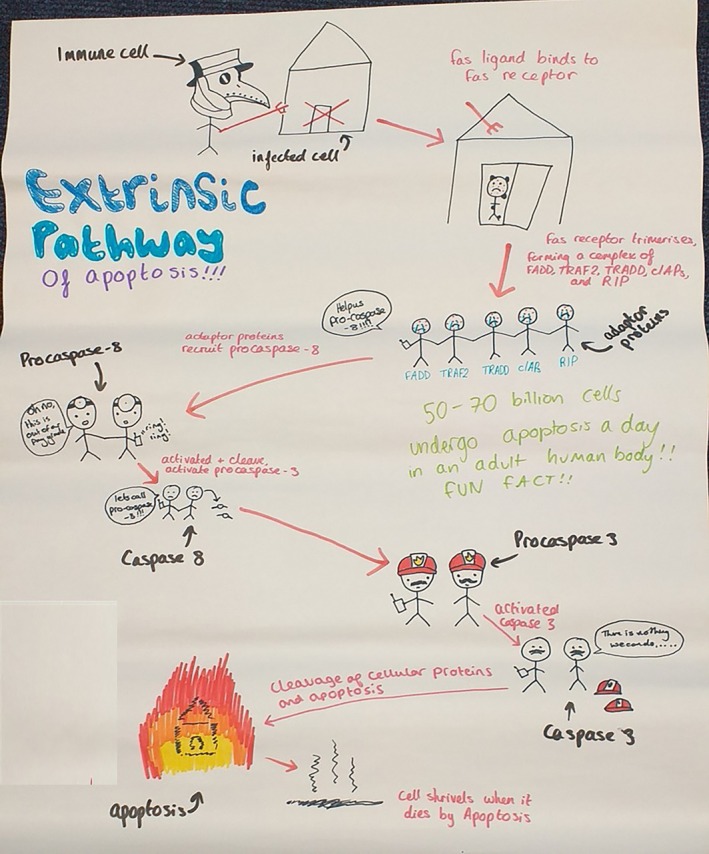
Exemplar of student‐generated concept cartoon illustrating the extrinsic apoptosis signaling pathway. An infected cell is represented by a marked house to indicate the presence of plague, the molecular components (adaptor proteins, caspase 8 and caspase 3) and their recruitment are represented by a series of calls to plague officials. Apoptosis itself is represented by a fire as an indication of an old practice where contaminated belongings and sometimes houses were burnt to stop plague spread.

**FIGURE 13:**
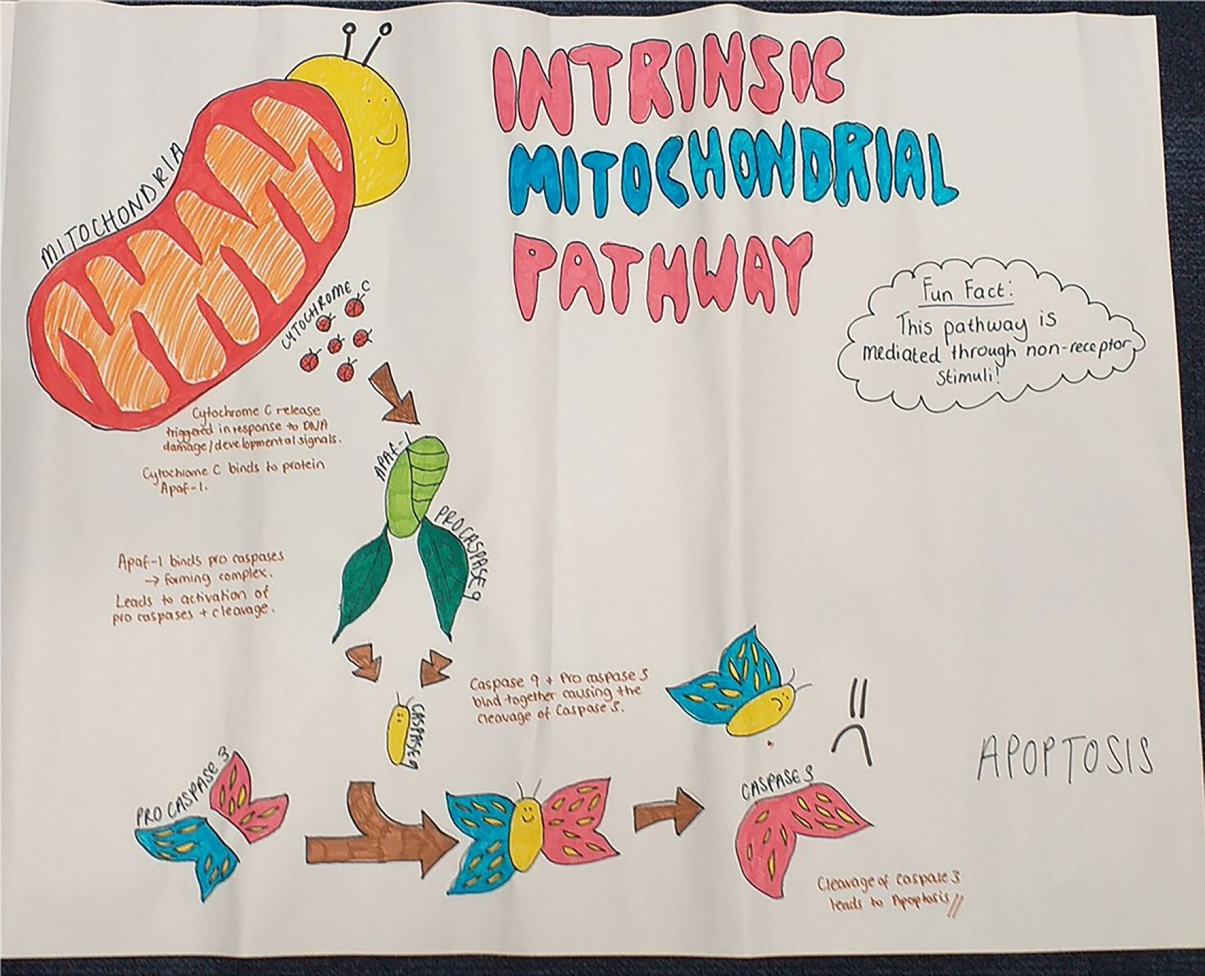
Example of student‐generated concept cartoon illustrating the intrinsic apoptosis signaling pathway. The caterpillar represents a cell with DNA damage. The chrysalis represents the stage of recruitment of Apaf‐1 and procaspase 9, the wings represent procaspase 3, and the adult butterfly with one wing represents the stage of apoptosis.

**FIGURE 14:**
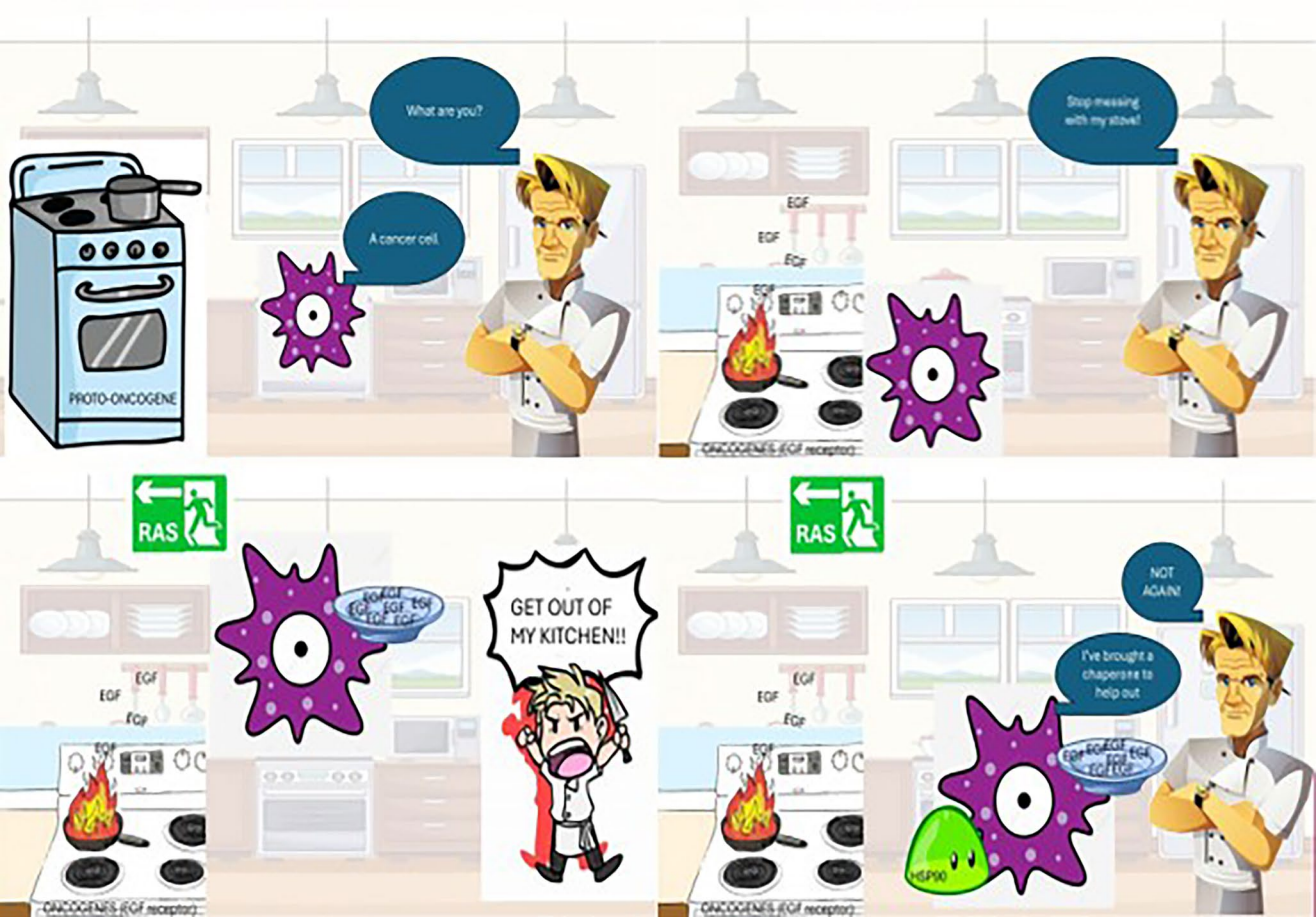
This group of students created a digital concept cartoon for self‐sufficiency in growth factors by illustrating a cancer cell commandeering Gordon Ramsay's kitchen to “cook its own food” in the form of epidermal growth factor (EGF).

**FIGURE 15:**
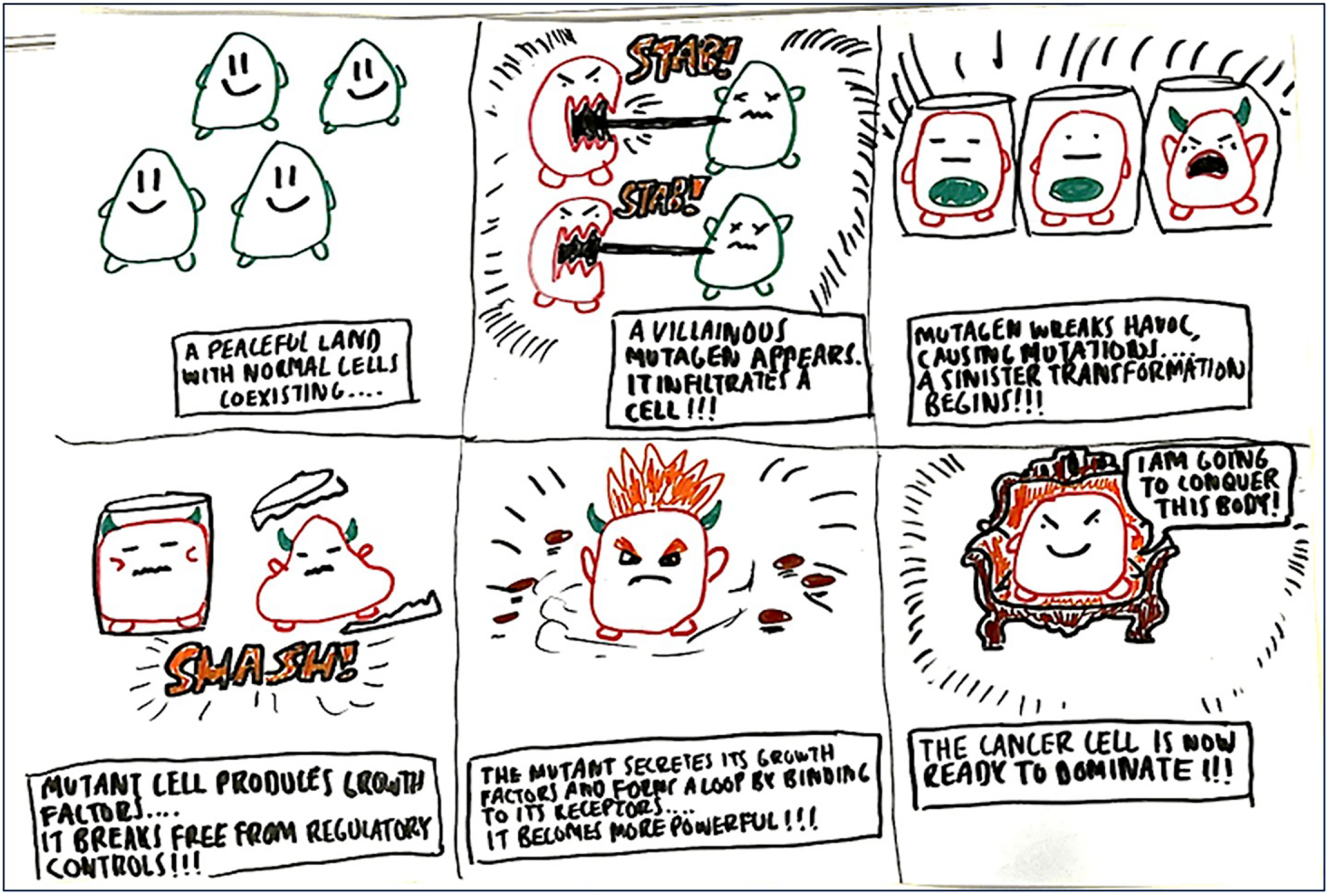
An alternative concept cartoon of the same hallmark, self‐sufficiency in growth factors, illustrates the transformation of healthy cells by a “villainous mutagen” into cancer cells that produce growth factors and break free from regulatory controls, secreting its own growth factors in an autocrine signaling loop.

Ethical approval to publish examples of students' cartoons was obtained from Loughborough University (2023‐16,192–16,306). In addition, students who participated in the activity gave informed consent to publish their work. Figures 10, 11, 12, 13, 14, 15 are examples of these cartoons.

## 4 Discussion

In this study, we invited students to work as a team in order to produce a cartoon‐style illustration of a complicated and multi‐step biology process. This is the first study to explore learning using concept cartoons created by students themselves, rather than ones designed by teachers. The quantitative and qualitative data collected through the survey show that there is a strong agreement that this cartooning activity offered a valuable learning experience which achieved the aims of the study.

The instructions and seminar design are presented in Figures 1 and 2. There was a strong agreement among students who took part in the cartooning activity that the seminar was well‐organized, the instructions were clear and that students would recommend the activity to other students (Figure 3). Hence, this study provides a framework for teachers willing to adopt a new learning and teaching strategy where students are tasked with constructing and reconstructing their knowledge on a specific topic. Although we conducted the seminars in three biology modules, the activity is translatable to other areas of study (this activity was conducted in the Horizons in STEM Higher Education Conference, University of Bristol, June 2024 where colleagues from various backgrounds took part). It is also worth noting that this activity was easy to set up, low cost, and did not require any specific technical skills.

### 4.1 First Aim: To Engage Every Student in the Content Beyond the First Level of Learning in Bloom's Taxonomy

According to Axelson and Flick [30], student engagement can refer to the extent to which learners are intellectually and emotionally invested in their educational experience as evidenced by their interest in the subject matter, their active participation in learning activities and their connection to their classes, institutions and peers. Although the authors question the possibility of measuring engagement accurately, they suggest that the following question can be asked: “How do we engage (cognitively, behaviorally, and/or emotionally) type X students most effectively in type Y learning processes/contexts so that they will attain knowledge, skill, or disposition Z? [30]”. This question strongly implies that the learning process is achieved via a shared responsibility between students and educational institutions (educators). Harrington et al. [38] emphasize that the relationship between students and staff is central for fostering engagement. Students are expected to exert the necessary effort to acquire knowledge and develop skills while educators are tasked with creating a suitable environment which enables student learning [30]. Via the cartooning activity, we aimed to effectively create a dynamic, interactive and student‐centered learning environment to foster engagement, collaborative learning and creativity. There was a strong agreement among students that the seminar offered a useful and engaging learning experience which encouraged them to participate in the class (Figures 3 and 4). Students commented on their improved attitude toward the subject matter after doing this activity (Figure 8). Furthermore, collaborative activity (including peer‐assisted learning) is proven to improve engagement [38, 39]. In this study, socialization, communication and group work constructed the collaborative learning experience in the cartooning activity (Figure 8). In addition, in the open‐ended responses there was a strong focus on engagement (Figures 8 and 9).

The hierarchy of the cognitive processes in the revised version of Bloom's taxonomy encompasses six categories progressing from simple to complex (remember, understand, apply, analyze, evaluate and create) [40]. The first level represented by the verb “remember” is associated with the cognitive function of recalling facts through listing, memorizing, repeating and stating [40, 41]. Via this cartooning activity, we aimed to involve students in higher‐order thinking based on the conceptual knowledge which was acquired in lectures [40]. By planning, creating and presenting the cartoons, students are expected to have shown:

- Understanding of the biological process through visuals and dialog in the cartoon (explaining the process using their own words).
- Application of knowledge through presenting the biological process in a new format/in a new situation (the story of the cartoon).
- Analysis through breaking down the biological process, exploring cause‐effect relationships and logically sequencing the stages of the biological process in the cartoon.
- Evaluation through decision making on what to include and to omit and how to simplify complex ideas so they are presented visually and in the story lines.
- Creativity through generating and producing an original cartoon strip that synthesizes their knowledge.

The quantitative and qualitative data both contain evidence of students' involvement beyond the first level of learning in Bloom's taxonomy. Data presented in Figure 5 shows that improved understanding, critical thinking, and identifying the main points in the taught topic scored on average 4.2, 4, and 4.3 respectively. Moreover, developing their creativity was one of the skills which students highlighted as a benefit from doing this activity (Figure 8).

### 4.2 Second Aim: To Give Every Student the Chance to Engage With an Emphasis on Building Knowledge and Skills

Acquiring soft skills and subsequently employability skills has been identified as a key aspect of students' higher education journey [42]. Communication skills, teamwork skills, leadership skills and innovativeness have been linked to improved performance in professional settings [43]. In addition, other skills including critical thinking, creativity, innovation, collaboration and resilience have been identified as necessary to succeed post‐graduation and provide relevancy to future job markets [44, 45]. Otermans et al. [42] explains that there is a skill gap between what students gain during their degree and what skills employers expect graduates to have. This gap has been linked to the fact that soft skills are being overlooked in educational and training programs [46]. A specific skill that has become an “educational imperative” for several reasons is creativity [47]. Despite the various unknowns surrounding the definition of creativity, how it should be taught and by whom and at which stages of education, there is a drive for viewing creativity a standard for education. Figure 8 shows that skill development was explicitly described by students as a benefit of doing the cartooning activity. In particular, students listed the following specific skills: collaboration, using technology, presenting, researching, creativity and confidence. It is highly important to note that the data related to skill development was mainly collected through the open‐ended responses rather than through answering specific questions in the survey. This strongly demonstrates that students independently reflected on their own learning process and articulated the impact of the cartooning activity. This explicit articulation of skill development makes soft skills visible and indicates students' understanding of the value of these skills as well as the recognition of the opportunities to develop them in academic activities.

### 4.3 Third Aim: To Create a Memorable Learning Experience With Long‐Lasting Impact Using a Student‐Centered Approach

When students were encouraged to draw concepts as cartoons, they worked to translate abstract biological processes into visual metaphors, processing and reconstructing the idea into the new medium. Cognitive psychological theories suggest that when information is presented in both verbal and visual formats (dual coding theory (DCT)) [48, 49] memory retention increases. Or that when working memory becomes limited it can impede coding and retention of information (cognitive load theory (CLT)). The limitations of working memory can be circumvented by organizing knowledge visually that in turn enables students to work on complex material without exceeding their cognitive capacity [50]. Thus, concerning cartoons, both CLT and DCT theories hold significance and offer potential mechanisms of action. From the data provided in this manuscript, cartoons acted as strong cognitive anchors for our students, providing distinctive, often humorous or exaggerated images (e.g., in Figure 9 linking complex terms such as “exonuclease” when cutting DNA as an “Ouch”), therefore helping long‐term recall by providing a semantic + visual + emotional memory cue. The use of visual media to enhance cognitive retention in relation to the STEM subjects has been seen for other visual co‐created materials such as cognitive maps [51, 52] working to reinforce the data presented herein.

The cartooning student‐centered seminar clearly moved away from didactic delivery and positioned the students as co‐creators of knowledge. By drawing cartoons themselves, students took ownership of their understanding, deciding which features were essential and how best to represent them; thus, the activity was student‐led [53, 54]. The theoretical literature on student‐centered learning (SCL) in higher education focuses on five aspects regarding the role of the teacher, the function of content, the responsibility for learning, the purpose of evaluations, and the balance of power [55, 56].

### 4.4 Concept Cartooning Student‐Centered Learning (CCSCL)

- Role of the teacher—Cartoon and content guide, act as the facilitator of learning.
- Function of content—to contribute to the learning process and acquisition of key skills rather than just memorization of biological cascades as “lists.”
- Responsibility for learning—shared between students and facilitator (teacher).
- Purpose of the evaluation—not only to generate grades but also to be a means for students to learn, practice skills, and give and receive feedback (needs to be clearly communicated).
- Balance of power—dynamic and changeable based upon pedagogical leanings of facilitator/teacher.

One aspect that was not fully evaluated within this study was the balance of power and this requires further consideration when moving forward with “concept cartooning” SCL [57]. The balance of power in SCL is important to evaluate because shifting authority from teacher to student can empower learners to take ownership and engage more deeply, but if not managed carefully, it may create confusion, uneven participation, or gaps in guidance. Generating the correct balance, through individual instructor awareness and effective management, is key to ensuring that autonomy enhances learning without compromising structure or academic rigor [58].

In the absence of additional seminar time, we suggest adopting a guided hybrid homework approach. This could involve providing students with clear examples and prompts and optionally allowing brief group discussions before assigning a structured out‐of‐class cartooning task to complete prior to the next session. Our experience indicates that some level of guided support, particularly during students' initial exposure to cartooning as a learning consolidation method, is essential to maximize engagement and optimize learning outcomes.

## 5 Conclusion

In this study, we provided educators with a framework for an activity which supports students to learn and understand a complex multi‐step process in a fun and engaging atmosphere. The activity promoted deep learning and skill development based on students' comments and feedback. Overall, tasking students with creating cartoons from scratch proved to be an effective method in making abstract and complicated topics more approachable.

## Funding

The authors have nothing to report.

## Ethics Statement

Ethical approval to collect data and to publish examples of students' cartoons was obtained from Loughborough University (2023‐16,192–16,306—LEON reference (LEON—Loughborough University Ethics ONline)).

## Consent

Students who participated in this study gave informed consent and voluntarily agreed to take part in this study.

## Conflicts of Interest

The authors declare no conflicts of interest.

## Data Availability

The data that support the findings of this study are available from the corresponding author upon reasonable request.
